# Citizens of somewhere: Examining the geography of foreign and native-born academics’ engagement with external actors

**DOI:** 10.1016/j.respol.2018.11.008

**Published:** 2019-04

**Authors:** Cornelia Lawson, Ammon Salter, Alan Hughes, Michael Kitson

**Affiliations:** a School of Management, University of Bath, Bath, United Kingdom; b Imperial College Business School, London, and Lancaster University Management School, Lancaster, United Kingdom; c Judge Business School and Centre for Business Research, University of Cambridge, Cambridge, United Kingdom

**Keywords:** Academic engagement, Foreign-born and native-born scientists, National collaboration, International collaboration

## Abstract

This paper explores the geography of academic engagement patterns of native and foreign-born academics, contrasting how patterns of intranational and international engagement with non-academic actors differ between these two groups. We suggest that foreign-born academics will engage more internationally than their native-born colleagues, whereas native-born academics will have greater levels of intranational engagement. Drawing upon a large multi-source dataset, including a major new survey of all academics working in the UK, we find support for the idea that where people are born influences how they engage with non-academic actors. We also find that these differences are attenuated by an individual’s intranational and international experience, ethnicity and language skills. We explore the implications of these findings for policy to support intranational and international academic engagement.

## Highlights

• The article provides new survey data on non-academic engagement activities of over 14,000 UK academics.
• We compare intra- and international engagement activities of foreign-born and UK-born academics.
• Foreign-born academics are relatively more engaged internationally but less intranationally than UK-born academics.
• Intranational differences diminish with years spent in the UK but foreign-born keep their international engagement premium.
• Language and ethnicity matter: foreign-born from a non-English or non-white background engage less intranationally.

But we also value something else: the spirit of citizenship. That spirit that means you respect the bonds and obligations that make our society work. That means a commitment to the men and women who live around you, who work for you, who buy the goods and services you sell.…But today, too many people in positions of power behave as though they have more in common with international elites than with the people down the road, the people they employ, the people they pass in the street. But if you believe you’re a citizen of the world, you’re a citizen of nowhere. You don’t understand what the very word ‘citizenship’ means.

Prime Minister Theresa May, 5 October 2016, Conservative Party Conference Speech.

I want this United Kingdom to emerge from this period of change stronger, fairer, more united and more outward-looking than ever before. I want us to be a secure, prosperous, tolerant country - a magnet for international talent and a home to the pioneers and innovators who will shape the world ahead. I want us to be a truly Global Britain – the best friend and neighbour to our European partners, but a country that reaches beyond the borders of Europe too. A country that goes out into the world to build relationships with old friends and new allies alike.

Prime Minister Theresa May, 17 January 2017, Lancaster House Speech on Brexit.

## 1 Introduction

In a bid to justify investments in science, governments across the globe are moving towards more mission or impact oriented funding, which requires researchers to contribute more towards public concerns, often of national scope. The focus on national issues and challenges is expressed in the first quote above, where UK Prime Minister Theresa May highlights the view that some members of society have greater alignment with, and interest in, people and problems from outside the nation than within it. The concern about the lack of domestic national engagement of ‘people in position of power’ does not explicitly mention academics, but it is clear that academics have a strong international orientation. For example, over half of the papers published by UK based academics in 2013 had an international co-author (Witze, 2016) and 30 per cent of the academic staff at UK universities are non-UK nationals (HESA, 2017). As it stands, the academic sector is one of the most globally oriented components of the national economic system. The second statement clearly acknowledges this and identifies the desirability of a country acting as a magnet for, and home to, international talent on a global scale. The global focus of academia is part of a longer tradition within academe of seeing academics as being ‘extra-territorial’, adhering to a set of norms, responsibilities and expectations that are established between academics without reference to national boundaries, rather than by adherence to distinctive national norms (Polanyi, 1962). In this sense, academics can be seen as being citizens of Polanyi’s metaphorical ‘republic of science’ alongside their national identities.

The Janus like approach in current politics represented by these speeches - and similar developments in the USA and Europe (Mazzucato, 2018; National Academies, 2010; National Research Council, 2014) - raises important issues for science policy and the geographical location and attraction of academics. If science policy is focused on attracting the best talent irrespective of nationality for the reasons set out in the second speech, what does this imply about the claimed need to focus on people and problems within the national context?

This paper addresses this question of the balance between the national and international by comparing the geography of academic engagement with policy and practice by foreign and native-born UK based academics. It is clear that collaboration and engagement between academics and non-academics is often shaped by geographic proximity (Breschi and Lissoni, 2001; D’Este et al., 2013; Ponds et al., 2010). Although prior research has highlighted differences between foreign and native-born academics in terms of scientific performance (Baruffaldi and Landoni, 2012; Franzoni et al., 2014; Jonkers and Cruz-Castro, 2013) industry engagement (Libaers, 2014), and the transfer of knowledge from returnees and expatriates (Edler et al., 2011; Trippl, 2013), we know relatively little about how the country of birth of an academic shapes the geography of their external engagements. In particular, we are unable to answer the following questions. *Do foreign and domestic-born academics differ in the ‘places’ in which they engage? If so, how do these differences manifest themselves? Do foreign-born academics pay less attention to ‘the people down the road’ than their native-born colleagues? Are these differences magnified or attenuated by an individual’s background and experiences?*

To explore these questions, we draw upon a rich, large, multisource dataset of UK academics, including a survey of channels of academic engagement with business, government and non-governmental organizations that arise out of an academic’s work, including: technology transfer; collaborative research; student projects; and policy advice (Bozeman et al., 2013; Perkmann et al., 2013). We begin by suggesting that foreign-born academics are liable to have higher levels of global engagement, as they are tapping into their rich international networks and relationships. In contrast, we argue that native-born academics display a greater intranational orientation in their engagement efforts, building on their knowledge and experience of the national context. We also probe how personal experience and background attenuate this relationship, highlighting intra- and international work experience, ethnicity and language skills. First, we argue that the longer foreign-born academics have resided in a particular national context, the more closely they resemble native-born academics in terms of their intranational orientation, without sacrificing their access to international networks. Moreover, we argue that native-born academics that have been educated or worked abroad will exhibit higher rates of international engagement. Second, we suggest that the ethnic background of academics will shape their intra- and international and engagement, with foreign-born academics with non-majority identities having greater differences in terms of intranational (lower) and international (higher) engagement than their native-born colleagues. We also suggest that native-born academics with non-majority ethnic identities exhibit higher rates of international engagement than their majority group domestic colleagues. Third, we argue that language skills play a role in the observed pattern of foreign-born academic engagement, with those individuals operating in their non-native language more likely to engage internationally. Overall, we find considerable support for these expectations and offer our interpretation of them in relation to the opposing viewpoints set out in the two speeches we have highlighted.

This paper makes three contributions to the literature. First, the study demonstrates that where academics are born has an impact on the geography of how they engage with non-academic stakeholders. In doing so, we bring attention to the way nationality shapes academic engagement, an issue that has received only modest attention thus far. Second, by demonstrating that foreign-born academics are relatively more engaged internationally whereas native-born academics are relatively more engaged intranationally, we contribute to a richer understanding of the geography of academic engagement and the balance between local, national and global connectivity. Third, by exploring how these differences are shaped by personal experience and characteristics, we shed light on some of the factors that drive these observed behaviours. We provide a rich and nuanced picture of academic engagement, which considers where people come from and their experiences. This approach also helps to build insights into how the career pathways of academics shape their external engagement, enriching our understanding of the micro-foundations of these behaviours.

## 2 Academic engagement with external actors

Academic engagement with external actors has become a broad and diverse research stream, examining the antecedents and consequences of these engagement patterns on academic careers and behaviours (Bozeman et al., 2013; Perkmann et al., 2013). In this context, academic engagement is understood as “knowledge-related collaboration by academic researchers with non-academic organisations” (Perkmann et al., 2013). It involves both formal activities, such as consulting, collaborative research, contract research, training, secondments, and informal activities, such as advice, networking, conference participation etc. Although most of the literature has focused on academic engagement with industry, there is an increasing interest in engagement with governmental and non-governmental organizations (Hughes et al., 2016; Landry et al., 2001; Olmos-Penuela et al., 2014). Within this literature, there has been an attempt to uncover the factors that led academics to engagement with non-academic actors. Research has shown that academic engagement is associated with: work experience in industry (Lin and Bozeman, 2006); high academic rank (Boardman and Ponomariov, 2009; Link et al., 2007); supportiveness and reward system of the university and department (Lach and Schankerman, 2008; Siegel et al., 2003); the behaviour of peers (Bercovitz and Feldman, 2008; Tartari et al., 2014); personal attitudes towards knowledge exchange (Boardman and Ponomariov, 2009; D’este and Perkmann, 2011); and the quality of the academic and their department and university (Perkmann et al., 2011; Ponomariov, 2008).

Research has addressed the geographical dimensions of academic engagement, with a strong focus on how distance shapes patterns of university-industry collaboration. The literature shows that geographic proximity, typically within a country, is a common feature of engagement and collaboration. Much of the literature has focussed on externalities with many studies emphasising the importance of local knowledge spillovers (Acs et al., 1992; Adams, 2002; Anselin, 2003; Jaffe, 1989; Ponds et al., 2010). For instance, Mansfield and Lee (1996) showed that large US firms not only cited universities within the US more often than those outside, but that within the US they prefer to collaborate with geographically close partners (placed within 100 miles). But, as Boschma (2005) has argued, geographical proximity is not a necessary nor a sufficient condition for spillovers to occur. There are different forms of ‘proximity’ and the different forms of academic engagement with non-academic partners (D’Este et al., 2013).

Using address data for publications with at least one address in the Netherlands, Ponds et al. (2007) demonstrated that university-industry collaboration tends to be more localized than academic collaboration, which is highly international, as geographical proximity may ameliorate institutional differences. This is also confirmed by Trippl (2013), who shows that, although a majority of internationally mobile academics maintain links with their former scientific community, only a minority have regular interaction with firms located there. Within the UK, Abramovsky and Simpson (2011) show that science-based firms are liable to locate close to universities with departments, especially high quality departments, in related areas of science. However, in many other industries they find little evidence that geographic proximity shapes engagement with universities. D’Este et al. (2013) show that the proximity effect is weakened when the firm is a member of a dense, technologically complementary cluster. In a related study, Laursen et al. (2011) examined the link between university quality and the geographical distance between universities and firms, finding that firms prefer collaborations with distant (including international) high quality universities over collaborations with low quality universities located nearby. Moreover, Broström (2010) shows in a survey of Swedish firms that geographic proximity matters when research collaborations focus on short-term immediate outcomes, but firms are liable to collaborate with international universities when focused on explorative, long-term efforts.

In summary, these studies suggest that the role of geographic proximity in enabling engagement between academia and industry is varied and subject to important limitations and qualifications. First, many studies focus on co-location (where economic actors are) and do not directly address the spillover mechanisms (what economic actors do). Second, studies that focus on regional production functions are limited by data availability and methodological limitations that subsume individual decision-making (Kirzner, 1966). Third, those studies that focus on the engagement activities of academics need to account for the diversity in the sector such as the quality of the academic’s department and university, the skills and knowledge of the industrial partners themselves and the nature of the collaboration. In this context, the impact of the nationality or country of origin of an academic on their engagement with external stakeholders has received only limited attention in the literature. This is particularly surprising given the importance of proximity in the literature reviewed above and the international nature of academia (Stephan, 2012).

## 3 The influence of country of origin on academic engagement

A key study looking at country of birth and academic engagement by Libaers (2014) explores the propensity of foreign-born academics to engage with industry in the US using a survey of scientists and engineers. Libaers finds that foreign-born academics are less likely than native-born academics to be approached by industrial firms for research collaboration, consultancy or joint technology commercialisation. However, they were more frequently co-authors with private firms on scientific articles (Libaers, 2014). The later finding was considered to be partly due to foreign-born academics’ focus on strong research performance, as they are active in the international labour market and need to perform well to sustain their immigration status (Libaers, 2014). This expectation is consistent with Stephan (2012), who argued that foreign-born scientists display high motivation and are subject to higher levels of selection than native-born scientists. Along these lines, Franzoni et al. (2014) suggest that foreign-born scientists might feel greater pressure to sustain their scientific performance than native-born scientists, as they are liable to lose their right to remain in their host country without secure employment.

In a second key study, Trippl (2013) investigates the industry engagement of internationally mobile and non-mobile star scientists using a sample of the most cited authors in the Web of Science. She shows that foreign-born star scientists do not engage less with firms intranationally compared to non-mobile scientists and thus concludes that they do not differ in their embeddedness. Trippl (2013) also argues that internationally mobile scientists create knowledge links between countries including between firms.

Extending on these prior efforts, we start with the view that the geography of academic engagement of foreign-born academics may differ to that of native-born academics. First, it is clear that the social capital of academics can have a significant influence on their engagement with non-academics (Lam, 2007). In particular, research has found that individuals with backgrounds of working in industry are more likely to engage with non-academic actors (Lin and Bozeman, 2006; Link et al., 2007). In the case of foreign-born academics, it may be that a lack of intranational social capital limits their ability to effectively engage with intranational non-academic actors. They are liable to lack the requisite networks and relationships to know whom to turn to outside of their university. In contrast, native-born academics will be able to draw upon their prior intranational social capital, accumulated during a lifetime of social interactions within and outside academia. At the same time, foreign-born academics are liable to have richer international social capital than their native-born colleagues, as they can draw upon relationships with colleagues and friends in their home country (Fernando and Cohen, 2016). This international social capital may open up possibilities for academic engagement outside the national context (Trippl, 2013).

Second, differences might also arise from contrasting research orientations. Foreign-born researchers are liable to have a non-local research orientation, attempting to tackle scientific problems that are not necessarily geared towards national missions. In contrast, native-born staff may be tempted to frame their research in terms of their home nation’s needs and problems, given their lack of exposure to the issues and problems faced by non-local actors and contexts (Jonkers and Cruz-Castro, 2013). Accordingly, foreign-born academics have been shown to collaborate with academics from a larger number of countries compared to natives (Scellato et al., 2015). In effect, foreign-born academics are liable to display a weaker attachment to the ‘place-based’ needs and issues of their host country than their native-born colleagues. This, in turn, makes it less likely that they will turn to intranational external actors to facilitate the development, conduct and exploitation of their research compared to native-born colleagues.

Third, foreign-born academics are liable to lack intranational, institutional knowledge – knowledge about ‘the way things are done around here’ - compared to their native-born colleagues. This lack of intranational institutional knowledge may make it harder for them to find appropriate partners for intranational collaboration and also to identify key contact points for intranational engagement with organisations. This is often a factor that acts as a key barrier to engagement itself (Tartari et al., 2012). Moreover, foreign-born academics may perceive fewer rewards in investing their time in intranational engagement since they are more aligned to international labour markets than their native-born colleagues (Libaers, 2014).

In summary, due to inadequate social capital, weaker attachment to place and limited institutional and organisational knowledge, foreign-born academics may suffer from a ‘liability of foreignness’. This leads them to engage with a more limited range of intranational external actors. In contrast, native-born academics with their intranational social capital, heightened sense of place and relatively lower international knowledge and experience may suffer from a ‘liability of domesticity’, leading them to engage less with international external actors. Thus,

H1a Foreign-born academics will exhibit greater levels of international engagement with non-academic organisations than their native-born colleagues.

H1b Native-born academics will exhibit greater levels of intranational engagement with non-academic organisations than their foreign-born colleagues.

## 4 Contextual factors

We have hypothesized about a number of generic differences that might be expected between foreign and native-born academics. It may be expected, however, that, given nationality, an individual’s personal experience and characteristics might weaken (or heighten) these observed patterns. In particular, the degree to which an individual ‘fits’ into their current national context might weaken the effect of where they were born, or their liabilities of foreignness and domesticity. We focus on three dimensions of an individual’s personal experience and background that might shape these differences: time spent intra- and internationally; ethnic background; and language skills. Below, we consider each of these factors in turn.

### 4.1 Time spent intra- and internationally

Working in a particular academic system will provide foreign-born individuals with a period of socialisation that will help to anchor them into the national context and this will help to overcome their liability of foreignness. Over time, they will have the opportunity to build richer and broader intranational social capital, often through interactions with colleagues, students and other institutional actors in the country. These ties may be developed and sustained through attending national meetings or conferences, engaging in collaborative research efforts and research events. Moreover, as they build up intranational professional experience, individuals will have a greater awareness of the expectations and requirements of the institutions that allocate research resources, such as funding agencies. They will also have more opportunities to find willing collaborators in industry or in government, helping them to craft strategies for effective resource mobilization from these intranational actors (D’este and Perkmann, 2011). They will also gain a richer understanding of the subtle institutional norms and ways of working (often only partially codified or understood by these actors), which can inhibit university-industry exchanges (Bruneel et al., 2010). Indeed, by working within the national context, researchers may find themselves drawn to more ‘place-based’ research problems or questions. In part, this shift in attention may be due to the funding requirements of national funding agencies for intranational engagement. It may also be due to the greater visibility of these problems to the researcher. As a result, as the time foreign-born academics spent working in the host country increases, differences between foreign and native-born academics will be expected to diminish.

H2a The difference between native- and foreign-born academics for intranational engagement will diminish with the time that foreign-born staff have spent working in the national context.

We suggest that native-born academics that have worked outside their home country might diminish their ‘liability of domesticity’ with respect to international engagement. Working in different international contexts might enrich their international social capital, allowing them to develop strategies to effectively engage with international actors in their research (Scellato et al., 2015). In addition, foreign experience will help them gain awareness and insights into scientific and technical challenges that differ from those in their home country (Gibson and McKenzie, 2014; Jonkers and Cruz-Castro, 2013). Finally, working in other national contexts will allow them to build up an understanding of the actors in these contexts and help them to frame research and engagement efforts in ways that appealing to the latter. As Edler et al. (2011) show, when scientists travel abroad for research they form ties with industry, as well as enhancing their scientific networks. Thus:

H2b The difference between native and foreign-born academics for international engagement will diminish if a native-born academic has worked outside their home country.

### 4.2 Ethnic background

We also suggest that the ethnic background of an academic may shape the geography of their academic engagement efforts. We expect ethnic background to matter for both foreign-born and native-born academics as it may pose both challenges and opportunities for intra- and international engagement. In the case of foreign-born academics, being a member of a non-majority ethnic group of the focal country can create significant additional layers of ‘foreignness’. This may stem from ethnic biases within the focal country. For example, research has shown that individuals with identical resumes with *non-majority* sounding names are five times less likely to receive a call back from human resource recruiters than individuals with resumes with *majority* sounding names (Kang et al., 2016). In the case of academic engagement where informal, face-to-face interactions are often the norm (Grimpe and Fier, 2010; Link et al., 2007), these biases may make it harder for non-majority foreign-born academics to find collaborative partners than their majority foreign-born or native-born colleagues.

The literature has also provided evidence for ethnic co-authorship and knowledge flows (Agrawal et al., 2008; Freeman and Huang, 2015) which is linked to the tendency towards homophily – the attraction to people like one’s self – in network formation (McPherson et al., 2001). A second challenge is thus related to the dearth of non-majority ethnic groups among senior roles and professions within the focal economy (Stephan, 2012). This means that non-majority foreign-born academics often have to forge ties with people who differ from them in terms of both nationality and ethnic background. In contrast, although they may lack intranational knowledge and social capital, foreign-born academics from the dominant ethnic background of the focal country are liable to find it easier to make contacts with intranational external actors, as they are less likely to face such subtle biases. Thus,

H3a The difference between native- and foreign-born academics in terms of intranational engagement will be greater if the foreign-born academic is a member of a non-majority ethnic background.

The case of native-born academics from non-majority ethnic backgrounds offers a different perspective. These individuals, when operating within their home national context, have the full advantage of language and deep contextual knowledge. However, the effects of their ethnic identity on their engagement may lead to enhanced international engagement. This is due two factors. First, non-majority populations often have a strong sense of attachment and identity to their country of family origin (Agrawal et al., 2011). These feelings of attachment might be reflected in the way these individuals organize their research; choosing research topics that are aligned to problems and challenges that are present in their family’s country of origin or by facilitating the family’s country of origin’s access to intranational knowledge (Agrawal et al., 2011). Second, when these individuals reach out to international collaborators in countries of their family’s origin, or their wider diaspora, they are able to draw on richer international social capital and knowledge than majority native born academics are likely to possess (see also Hegde and Tumlinson, 2014; Rauch and Trindale, 2002). As such, they may be more effective at finding partners and collaborators from outside the focal country. Thus, we would expect that the ethnic backgrounds of native-born academics to shape the geography of their academic engagement, leading individuals from non-majority backgrounds to have greater international engagement than their majority colleagues.

H3b The difference between native- and foreign-born academics in terms of international engagement will diminish if the native-born academic is from a non-majority ethnic background.

### 4.3 Language skills

Many foreign-born academics face the challenge of working in their second or even their third language. This may lead to linguistic hurdles in effectively reaching out to industrial, government or non-governmental partners in their engagement efforts. In contrast, foreign-born academics with the same native language as the native-born will not experience these linguistic hurdles (Bhaskar-Shrinivas et al., 2005). One may believe language ability to provide less of an advantage in the context of the UK compared to countries with less common languages, however language ability has been shown to be more important for building inter-personal relationships in English-speaking countries (Bhaskar-Shrinivas et al., 2005).

H4a The difference between native and foreign-born academics in terms of level of intranational engagement will be heightened if the foreign-born academic comes from a non-native English speaking country.

These intranational disadvantages might turn into advantages in the case of international engagement, where the ability to speak the local language is liable to help facilitate engagement with external actors (Bhaskar-Shrinivas et al., 2005). In contrast, native-born academics may lack fluency in other languages and be inhibited in developing an international orientation. For example, Gibson and McKenzie (2011) find that, amongst highly able students in a pacific island countries, those who did not study a foreign language were less likely to emigrate. This may be also be a specific feature of the UK, as the ability to speak a foreign language in the UK is significantly lower than many other European countries (EC, 2012). As a result, UK-born academics are liable to be less likely to build relationships with international actors who may only have partial working knowledge of English. Thus:

H4b The difference between native and foreign-born academics in terms of the level of international engagement will be heightened if the foreign-born academic comes from a non-native English speaking country.

## 5 Research context and data

Our study is based on the individuals working within UK academia, which is a large complex system with a distinctive funding structure, an outstanding academic performance record and a large and rapidly growing share of foreign born staff. There are over 160 higher education institutions in receipt of public funding for teaching and or research in the UK^1^. These institutions are independent self-governing not-for-profit charitable foundations with substantial research funding from public sources through the “dual funding system”. Of the additional funding streams, which include Private Not-for-Profit charitable sources the private business sector and overseas sources, the latter have been the only significant group to show an increase in real terms since 2008/9. This reflects the distinctively open international nature of the funding of UK public and private sector R&D by international standards (Hughes and Mina, 2012).

There is a wide range of different types of universities, including specialist institutions in the creative and performing arts, which vary in terms of disciplinary focus, research intensity age and mission. Typically, UK universities are categorised into three groups: 1) 24 research-intensive universities of the Russell Group; 2) so called ‘red brick’ or ‘plate glass’ universities of the 1920s and 1960s education expansion respectively; and 3) ‘post-1992’ universities which are former polytechnics that converted to university status in reforms enacted in 1992.

In 2015/16, UK universities employed around 195,000 academic staff with teaching and or research duties. Decision-making about employment resides with individual universities and they have been highly successful in recruiting international academics. As a result, the number of foreign-born nationals in the system is high by international standards (Scellato et al., 2015) and they account for a relatively high share of faculty at leading research-oriented universities. Indeed, foreign nationals accounted for over 60% of the total growth in academic staff numbers since 2006/7 (UniversitiesUK, 2016, 2017). The UK system also remains highly productive in terms of research outputs. Although representing only 4 per cent of the world total of academic researchers, it accounts for around 16 per cent of the world’s most highly cited articles (UniversitiesUK, 2017).

Since 2007, the UK Government has formally promoted an ‘impact agenda’ to reward and encourage academics (and their universities) to engage with non-academic audiences (Martin, 2011). Similar pressures for impact or mission-oriented research have manifested themselves in the USA (National Academies, 2010; National Research Council, 2014), and in the rest of Europe (LERU, 2017; Mazzucato, 2018). Within the UK context, this ‘impact agenda’ is reflected in funding for knowledge exchange for universities, through the REF itself, the research council’s requirement for a ‘pathway to impact’ for all grant applications, and direct funding for universities’ knowledge exchange efforts. Academic contacts with external organisations along these pathways have this become an important area both for research and policy (Deiaco et al., 2012). Given the open nature of the UK university system and these contextual factors emphasising impact, this context provides a rich environment to study external engagement patterns across native and foreign-born academics.

To examine our research questions, we make use of a large-scale survey of academics in the UK conducted by the Centre for Business Research (CBR) in 2015 (Hughes et al., 2016). The survey targeted all academics active in teaching and/or research in all academic fields and at all universities in the UK. Academic staff were identified from university departmental websites and their email addresses were collected by hand. This resulted in a sample of approximately 140,000 academics with known email addresses to which a web-based survey was sent. Of the emails sent, 8422 were undeliverable due to outdated contact details. Complete responses were received from 18,177 academics (13% response rate). A detailed set of response bias tests (available upon request) show little or no bias and the dataset is thus a representative sample of the UK academic population.^2^ After removing respondents with missing values in responses of interest for the purpose of this study, along with respondents that are retired, in teaching-only contracts, or in research assistant positions, we are left with a final sample of 14,574 from 151 different universities.

The survey asked respondents about their engagement with external, non-academic, institutions in the pre-survey period from 2012 to 2015. It included questions on 27 different channels of engagement with a broad coverage of external organisations, including those in public or non-governmental organisations in addition to interactions with private sector firms. The survey asked respondents to indicate whether these interactions took place intranationally (in the UK) or internationally (outside the UK). The survey also included questions yielding data on a wide range of individual academic characteristics including age, gender, academic rank, disciplinary field, country of birth and of PhD award. It also asked about each respondent’s prior work experience, research orientation and career motivations. These features make the survey one of the largest and most comprehensive micro-level datasets available for any economy that provides data on the engagement of academics with non-academic actors.

The survey data is complemented with information from other individual, institutional and regional level datasets: 1) information from the research councils to establish those academics that held research council funding during the 2012 to 2015 period; 2) university-level research contract income from the Higher Education Statistics Agency (HESA) for the academic year 2013/14; 3) university-level research quality scores from the 2014 REF as calculated by Times Higher Education (THE); and 4) R&D expenditure and population density by region from Eurostat.

### 5.1 Measures of foreign-born

The country of origin of each academic was determined by asking respondents about their country of birth and creating a binary variable *foreign-born* for those academics born outside the UK. The academic respondents were from 151 different countries with 35% being born outside the UK. This is consistent with prior surveys, such as the GlobSci survey which reported a share of foreign-born of 32% for the UK (Scellato et al., 2015). The largest group of foreign-born academics come from developed countries, such as Germany, the US and Italy (each comprising more than 400 respondents). However, there are also large numbers of academics from developing countries, including China, India, Pakistan, Nigeria and Iran.

To identify the ethnicity of respondents, we undertook two steps. First, we identified the likely ethnicity based on country of birth using the ethnocultural characteristics provided by the UN Statistics Division (2017). Second, we used the software tool Ethnea (Torvik and Agarwal, 2016), which maps names to 26 predefined ethnicities based on geo-coded first and last author names in Pubmed. We then compared likely ethnicity returned by the two methods and, where the two did not match, conducted additional web searches. This was the case for, for instance, those of British descent born in Zimbabwe or Hong Kong, of Indian descent born in Kenya or those of Chinese descent born in Australia. For the purpose of this study, we combined all ethnicities into two classes: white (i.e. of English, German, Italian, Hispanic, etc., ethnicity) and non-white (i.e. of Chinese, Arab, African, Indian, etc., ethnicity). And created two binary variables (*non-white foreign-born* and *non-white UK-born*), which are 1 if the academic is from a non-white ethnicity. In our sample, the proportion of non-white is 22% within the population of foreign-born (*non-white foreign-born*) and 2% within the population of UK-born (*non-white UK-born*). The low percentage of non-white within the UK-born population shows the difficulty of any name classification tool in completely picking up ethnicity within countries with an ethnically diverse population. For example, in the case for UK-born academics of Caribbean descent the name algorithm would assign ‘English’ as the ethnicity. It may also reflect the lack of diversity among the UK-born within the academic sector where only three per cent of department heads are members of a non-white ethnic group (Rathi and Ware, 2014).

Foreign-born respondents were further classified as either native English speaker or non-native English speaker according to the dominant language in their country of birth (UN Statistics Division, 2017) and a binary variable takes the value 1 for “*non-English native”* speakers. The proportion of respondents from non-English speaking countries amongst the foreign-born is 65%.

In addition to the country of birth, the subsequent experiences of academics in other national contexts should be expected to play an important role in determining involvement in intra- and international engagement of UK based academics. The survey did not directly ask about time spent in the UK or other countries. This was therefore inferred from answers to other questions. To quantify the time foreign-born academics spent in the UK, we used the following three survey questions: *Where did you receive your highest degree/qualification? How long have you been employed by your current HEI? Were you employed by another university immediately before you joined your current HEI and was your previous university a UK HEI*? These provide only a partial indicator of the number of years spent in the UK. We therefore firstly calculated the approximate number of years that passed since their PhD for those that completed their PhD in the UK. For those that completed their PhD elsewhere, we considered the number of years spent at the current institution and added additional years if they had prior UK employment experience. We then assign each to three groups: ‘*recent arrival’*, ‘*settled’*, and ‘*long-term settled’* according to the sample distribution. The variable *“Time in the UK”* takes the value 1 (recent arrival; 32%) for academics who spent less than 7 years in the UK; the value 2 (settled; 32%) for those who had spent less than 13 years but 7 or more years; and the value 3 (long-term settled; 36%) for those who had spent 14 or more years in the UK.

To identify those UK-born academics that spent some time abroad, we used data from two survey questions regarding the location of their PhD and the location of their prior employment. The variable “*returnees*” takes the value 1 for UK-born respondents who completed their PhD outside the UK or held their last academic position outside the UK. This is the case for 8% of native-born academics and it may underestimate the underlying level of international mobility of UK academics as it ignores migration at other career stages.

### 5.2 Dependent variable - patterns of engagement activity

Building on the prior literature (Link et al., 2007; Perkmann et al., 2013), the survey enquired about 27 channels of knowledge exchange with external organisations. For each channel, the survey asked whether they were undertaken intranationally (within the UK, including locally) or internationally (outside the UK). These two variables are not mutually exclusive and respondents were able to indicate that they performed both. In our main analysis, we only consider the 15 engagement activities most often reported by survey respondents in order not to skew the results towards less important forms of engagement.

We constructed an aggregate dependent engagement variable by summing the number of different engagement activities. This means that an academic with zero activities scores a 0, and one engaged in all activities scores 15. It can thus be considered a measure for engagement breadth (D’este and Perkmann, 2011; Laursen and Salter, 2006). We constructed this measure separately for intra- and international engagement. Both measures have a good degree of internal consistency (Crohnbach’s alpha of 0.80 and 0.83 respectively).

## 6 Analytical procedures

To estimate the association between country of birth and academic engagement, we adopt two different estimation strategies. First, we estimate a series of Poisson regression count data models that measure the number of channels used intranationally and internationally, while controlling for individual, department, university and regional characteristics. In particular, the models include controls for being female, age, academic rank (seniority), years at current institution and being in receipt of a research council grant. We also include a measure for intrinsic career motivation which is based on the average responses to the following question: “*When thinking about your job as an academic, how important is each of the following factors to you*?” on a 1–5 Likert scale (from “completely unimportant “to “very important”). These include intellectual challenge, independence, responsibility and contribution to society. We further include the academic’s research orientation classified as *basic*, *user-inspired*, *applied* or *none* and the disciplinary field. University controls include dummies for research-intensive Russell Group universities and for post-1992 universities, the universities’ external research income and the university level 2014 REF Research Output Score. The decision to engage may also be shaped by the regional environment captured by R&D expenditure at the NUTS-2 sub-regional level and population density at the NUTS-3 sub-regional level. We further include dummies for the devolved regions of the UK.

A second estimation strategy employs matching estimators (Heckman et al., 1997) which allow us to compare the engagement breadth of foreign-born academics to a closely matched UK-born peer. We use a semi-parametric matching method, which has the advantage over parametric models that it avoids assumptions about functional forms and error term distributions (Rosenbaum and Rubin, 1983). In addition, we reduce possible bias by combining the propensity score matching with elements of an exact matching (EM) procedure to avoid bad matches for important characteristics which may impact the observed differences. These are academic rank, disciplinary field, university and female. In absence of performance measures, such as publications, we adopt a very fine-grained disciplinary field matching (17 subfields) which ensures that academics are matched with peers within the same department and thus subject to the same promotion and evaluation criteria. We match each foreign-born in our population to a UK-born academic with similar characteristics, using a propensity score that summarizes a set of observable characteristics affecting the probability of being foreign. These are age, whether an academic received their degree in the UK, research orientation and research council funding receipt. We then calculate the Mahalanobis distance to select the closest neighbour. This procedure returns a match for 1451 foreign-born academics. After the matching procedure, we do not observe any significant difference in any of the covariates between the treated and the control group. Appendix Table A1 reports the propensity score estimation before and after the matching.

Using the matched comparison group the average treatment effect on the treated (foreign) researchers can be summarized as:

(1) $$\alpha^{TT}=E\left(Y^{T},T=1,\;X=x\right)-E\left(Y^{C},T=0,\;X=x\right)$$

where *Y^T^* indicates the set of engagement related activities of academics. The potential engagement *Y^c^* which would have been realized if the foreign-born group (*T = 1*) had not been foreign, is estimated from the control group of UK-born academics that have similar characteristics in *X* (Rosenbaum and Rubin, 1983). The average effect on the treated can thus be calculated as the mean difference of the matched samples:

(2) $$\alpha^{TT}=\;\frac{1}{N^{T}}\sum_{i=1}^{N^{T}}\left(Y_{i}^{T}-{\hat{Y}}_{i}^{c}\right)$$

with $\hat{Y_{i}^{C}}$ being the counterfactual for *i* and *N^T^* the sample size of foreign academics.

As we perform sampling with replacement to estimate the counterfactual situation an ordinary t-statistic on mean differences would be biased, as it does not take the repeated observations into account. To correct the standard errors, we follow Lechner’s (2001) procedure for an asymptotic approximation of the standard errors in order to draw conclusions on statistical inference.

Variable descriptions for all variables are reported in Table 1, which also indicates the data source and whether the variable was used in the matching.

**Table 1:** Variable description.

Variable name	Measurement	Source	Matching
**Dependent**			
intranational engagement	Number of different engagement activities with intranational external actors	Survey	
international engagement	Number of different engagement activities with international external actors	Survey	
**Foreign-born**			
Foreign-Born	Dummy variable taking value 1 for those born outside the UK	Survey	
*Characteristics of UK born*			
Returnee	Dummy variable taking value 1 for those born in the UK but with PhD or last employment outside the UK	Survey	
Non-white UK born	Dummy variable taking value 1 for those born in the UK and whose ethnicity is non-white	Survey, Ethnea	
*Characteristics of Foreign born*			
Time in UK	recent arrival: Spent less than 7 years in the UK settled: spent 7 to 13 years in the UK long-term settled: spent 14 or more years in the UK	Survey	
Non-white foreign born	Dummy variable taking value 1 for those of non-white ethnicity born outside the UK	Survey, Ethnea	
Non English native	Dummy variable taking value 1 for those born outside the UK with a language other than English as their mother tongue	Survey, UN	
**Other Variables**			
Female	Dummy variable taking value 1 for female academics	Survey	EM
Age	3 age categories: <40; 40-49, >50	Survey	p-score
Yrs employed at current HEI	Number of years employed at current institution	Survey	
PhD in UK	Dummy variable taking value 1 for those who completed their PhD in the UK	Survey	p-score
Academic rank	4 seniority categories: Professor; Reader, Associate, Senior Lecturer; Lecturer; Research Fellow, Associate	Survey	EM
Research orientation	4 categories: Basic; User-inspired; Applied; None	Survey	p-score
Career Motivation: Intrinsic	Average score of importance of *intellectual challenge, independence, responsibility* and *societal contribution* on 5 point scale from "completely unimportant" to "very important"	Survey	
Research Council Funding	Dummy variable taking value 1 for principal investigators on a research council grant 2012-2015	RCUK	p-score
Disciplinary field	4 field categories: social sciences; life science & health; arts and humanities; engineering, maths, physics	Survey	
	17 field subcategories		EM
University	151 universities	Survey	EM
University type	3 categories: post-1992; Russell Group; other	Survey	
University REF Research Output Score	University grade point average (GPA) on a scale from 0 to 4 based on REF 2014 results	Times Higher Education	
University research contract income	Amount of 2013/14 external research contract income per permanent academic staff	HESA	
NUTS2_R&D expenditure (2014)	Annual R&D expenditure within the region	EUROSTAT	
NUTS3_Population Density (2015)	Population density within the local area	EUROSTAT	
Region	4 categories: England; Northern Ireland; Scotland; Wales	Survey	

EM = Exact matching; p-score = propensity score; RCUK = Research councils UK.

Ethnea (Torvik and Agarwal, 2016); UN (UN Statistics Division, 2017).

## 7 Results

We begin by presenting a descriptive analysis then review the results from the Poisson estimation before we turn to the matching analysis.

### 7.1 Descriptive results

**Fig. 1:**
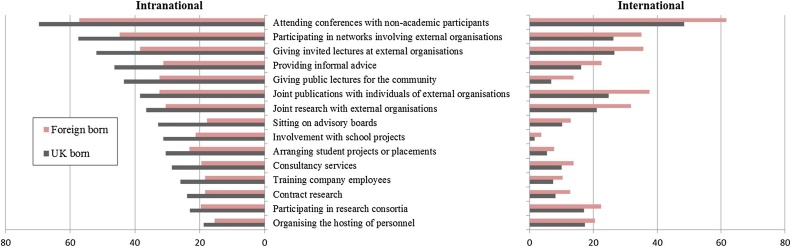
Share of respondents using each channel of engagement with external partners (in %). Note: All differences are statistically significant. 9415 UK born and 5159 foreign born respondents.

Fig. 1, reports the 15 activities and the share of UK and foreign-born respondents engaging in each intra-and internationally. The data on intranational interactions show that foreign-born academics are on average less likely to be involved in all 15 activities relative to UK-born academics. Exactly, the reverse is true for international interactions. It is also clear from Fig. 1 that both UK and foreign-born academics are much more frequently involved in intranational than in international interactions. The most widely undertaken intranational activities by both native and foreign-born academics are: conferences involving non-academics and network participation. In addition, over 40% of native-born academics were involved intranationally in invited lectures, public lectures and informal advice. For foreign-born academics invited and public lectures, joint publications and informal advice were the next most highly ranked activities (all >30%).

The relative frequency of types of international interactions is similar across foreign-born and native academics, though in all cases absolute participation is higher for the foreign-born. For both groups, the top five most frequent activities include conferences, network participation and invited lectures along with joint publications and joint research. This suggests that although foreign-born academics are *relatively* more likely than those who are native-born to interact internationally, they are *absolutely* more likely to be involved intra- rather than internationally.

### 7.2 Poisson results

Descriptive statistics of all regression variables are reported in Table 2 and the correlation between these variables is shown in Table 3. The foreignness variables generally show low correlation with other explanatory variables, with the exception of UK PhD.

**Table 2:** Descriptive statistics of regression variables (N = 14,574).

Dependent	mean	sd	min	max
Intranational engagement	5.10	3.48	0	15
International engagement	2.81	3.04	0	15
**Main explanatory**				
Foreign Born	0.35	0.48	0	1
*Characteristics of UK born*				
Returnee	0.05	0.22	0	1
Non-white UK born	0.01	0.11	0	1
*Characteristics of Foreign born*				
Time in UK = recent	0.11	0.32	0	1
Time in UK = settled	0.11	0.32	0	1
Time in UK = long-term settled	0.13	0.33	0	1
Non-white foreign born	0.23	0.42	0	1
Non English native	0.08	0.27	0	1
**Controls**				
Female	0.41	0.49	0	1
AGE < 40	0.32	0.47	0	1
AGE 40–49	0.28	0.45	0	1
AGE >49	0.39	0.49	0	1
Yrs employed at current HEI	7.83	5.37	0	15
PhD in UK	0.81	0.39	0	1
Professor	0.22	0.41	0	1
Reader, Associate Professor, Senior Lecturer	0.34	0.48	0	1
Lecturer	0.23	0.42	0	1
Research Fellow, Research Associate	0.21	0.41	0	1
Basic research	0.26	0.44	0	1
User-inspired basic research	0.26	0.44	0	1
Applied research	0.43	0.50	0	1
None of the above apply to my research	0.04	0.20	0	1
Career Motivation: Intrinsic	4.36	0.47	1	5
Research Council Funding	0.12	0.32	0	1
Social sciences	0.26	0.44	0	1
Life Science & Health	0.34	0.47	0	1
Arts and Humanities	0.16	0.37	0	1
Engineering, Maths, Physics	0.24	0.42	0	1
Post 1992 University	0.28	0.45	0	1
Russell Group University	0.46	0.50	0	1
University REF Research Output Score	2.82	0.38	0	3
University research contract income	3.56	1.29	0	6
NUTS2_R&D expenditure (2014)	9.70	10.15	1	34
NUTS3_Population Density (2015)	7.30	1.48	3	10
region NORTHERN IRELAND	0.02	0.14	0	1
region SCOTLAND	0.10	0.30	0	1
region WALES	0.05	0.22	0	1

**Table 3:** Correlation table of regression variables (N = 14,574).

		1	2	3	4	5	6	7	8	9	10	11	12	13	14	15	16	17	18	19	20	21
1	Intranational	1.000																				
2	International	0.356^*^	1.000																			
3	Foreign-born	−0.190*	0.149*	1.000																		
4	Returnee	−0.025*	0.030*	−0.170*	1.000																	
5	Non-white UK born	0.008	−0.004	−0.086*	0.031*	1.000																
6	Time in UK	−0.106*	0.150*	0.894*	−0.152*	−0.077*	1.000															
7	Non-white foreign born	−0.202*	0.124*	0.740*	−0.126*	−0.064*	0.615*	1.000														
8	Non English native	−0.067*	0.067*	0.396*	−0.067*	−0.034*	0.404*	0.271*	1.000													
9	Female	−0.027*	−0.134*	0.004	−0.036*	0.001	0.018	−0.009	−0.049*	1.000												
10	AGE < 40	−0.208*	−0.106*	0.217*	−0.006	0.030*	0.031*	0.217*	0.085*	0.048*	1.000											
11	AGE 40 - 49	0.058*	0.003	0.022*	−0.001	0.014	0.090*	0.018	−0.003	0.045*	−0.437*	1.000										
12	Yrs employed at current HEI	0.184*	0.094*	−0.248*	0.018	−0.027*	−0.064*	−0.234*	−0.106*	−0.072*	−0.545*	0.048*	1.000									
13	PhD in UK	0.189*	−0.114*	−0.532*	−0.137*	0.033*	−0.240*	−0.444*	−0.073*	0.048*	−0.153*	−0.007	0.203*	1.000								
14	Research Fellow, Associate	−0.179*	−0.060*	0.162*	−0.025*	0.025*	0.056*	0.168*	0.086*	0.068*	0.382*	−0.099*	−0.342*	−0.133*	1.000							
15	Lecturer	−0.099*	−0.144*	0.039*	0.002	0.009	0.013	0.026*	0.012	0.065*	0.190*	−0.003	−0.203*	0.000	−0.281*	1.000						
16	Reader, Associate Professor, Senior Lecturer	0.064*	−0.075*	−0.084*	−0.012	−0.000	−0.024*	−0.076*	−0.027*	0.023*	−0.204*	0.143*	0.210*	0.074*	−0.374*	−0.394*	1.000					
17	User-inspired basic research	0.019	0.060*	0.068*	−0.005	−0.003	0.063*	0.072*	0.027*	−0.020	0.002	0.009	−0.016	−0.039*	−0.032*	0.024*	−0.013	1.000				
18	Applied research	0.225*	0.100*	−0.106*	−0.056*	0.030*	−0.076*	−0.093*	0.017	0.060*	−0.038*	−0.008	0.015	0.130*	0.080*	−0.051*	0.000	−0.523*	1.000			
19	None of the above	−0.060*	−0.092*	−0.057*	0.007	−0.007	−0.039*	−0.055*	−0.037*	0.049*	−0.041*	−0.013	0.037*	0.034*	−0.053*	0.048*	0.045*	−0.127*	−0.184*	1.000		
20	Career Motivation: Intrinsic	0.169*	0.170*	0.066*	0.003	0.026*	0.086*	0.030*	0.023*	0.109*	−0.065*	0.030*	0.042*	−0.010	−0.094*	−0.034*	0.023*	0.037*	0.034*	−0.035*	1.000	
21	Research Council Funding	0.120*	0.145*	−0.037*	0.042*	−0.016	−0.016	−0.037*	−0.040*	−0.075*	−0.102*	0.048*	0.142*	−0.002	−0.127*	−0.104*	−0.030*	0.056*	−0.081*	−0.053*	0.047*	1.000
22	Life Science & Health	0.046*	0.000	−0.089*	0.004	0.030*	−0.087*	−0.078*	−0.053*	0.107*	0.018	0.011	0.009	0.035*	0.118*	−0.049*	−0.023*	−0.071*	0.150*	−0.059	0.017	−0.000
23	Arts and Humanities	−0.055*	−0.103*	−0.034*	0.016	−0.022*	−0.000	−0.064*	−0.068*	0.058*	−0.049*	0.036*	0.043*	0.044*	−0.109*	0.041*	0.053*	−0.035*	−0.188*	0.192*	0.016	−0.042*
24	Engineering, Maths, Physics	−0.014	0.118*	0.134*	0.037*	−0.003	0.078*	0.164*	0.112*	−0.224*	0.094*	−0.042*	−0.010	−0.149*	0.108*	−0.047*	−0.072*	0.059*	−0.037*	−0.079*	−0.063*	0.146*
25	Post 1992 University	0.077*	−0.128*	−0.122*	−0.055*	0.013	−0.069*	−0.106*	−0.003	0.056*	−0.121*	0.010	0.040*	0.152*	−0.220*	−0.011	0.295*	−0.028*	0.097*	0.051*	−0.012	−0.164*
26	Russell Group University	−0.087*	0.085*	0.110*	0.038*	−0.011	0.058*	0.091*	0.009	−0.057*	0.131*	−0.025*	−0.057*	−0.126*	0.240*	−0.060*	−0.212*	0.007	−0.075*	−0.049*	0.000	0.129*
27	University REF Research Output Score	−0.083*	0.106*	0.117*	0.039*	−0.004	0.070*	0.110*	0.021	−0.063*	0.109*	−0.017	−0.031*	−0.134*	0.181*	−0.046*	−0.187*	0.021	−0.076*	−0.049*	0.012	0.117*
28	University research contract income	−0.080*	0.164*	0.148*	0.054*	−0.011	0.084*	0.126*	0.021	−0.072*	0.150*	−0.029*	−0.053*	−0.172*	0.279*	−0.036*	−0.290*	0.013	−0.064*	−0.072*	0.014	0.170*

^*^p <  0.01; Correlations of other variables are omitted for space reasons. All show low correlations with main variables of interest.

Table 4 presents the results of the Poisson estimation testing for correlations between the foreign-born status of individuals and the breadth of their engagement. We use a base model that estimates the differentials in engagement with respect to UK-born academics. This is shown in Model 1 and includes the single “foreignness” variable *Foreign-born.* Model 2 includes measures for experience (*Time in UK* and *Returnee*) and for ethnicity (*non-white)* and language (*non-native*). In this model, the omitted status is white UK-born with no foreign experience.

**Table 4:** Poisson regression on number of used engagement activities (max 15).

	Intranational		International		Intranational		International	
	*dy/dx*	*se*	*dy/dx*	*se*	*dy/dx*	*se*	*dy/dx*	*se*
Foreign Born	−0.595***	0.067	0.975***	0.060				
*Characteristics of UK born*								
Returnee					−0.538***	0.144	0.701***	0.109
Non-white UK born					−0.190	0.228	0.369*	0.202
*Characteristics of Foreign born*								
Time in UK = recent					−0.958***	0.173	1.279***	0.173
Time in UK = settled					−0.027	0.136	0.930***	0.120
Time in UK = long-term settled					−0.016	0.105	0.861***	0.088
Non-white foreign born					−0.343***	0.123	0.065	0.081
Non English native					−0.948***	0.105	0.294***	0.074
*Controls*								
Female	−0.184***	0.055	−0.590***	0.051	−0.198***	0.055	−0.580***	0.051
AGE < 40	−0.268***	0.086	−0.192**	0.079	−0.197**	0.090	−0.248***	0.084
AGE 40-49	0.298***	0.065	0.003	0.059	0.310***	0.065	−0.011	0.059
Yrs employed at current HEI	0.015**	0.006	0.006	0.005	0.005	0.006	0.012**	0.006
PhD in UK	0.937***	0.096	−0.301***	0.064	0.334**	0.132	−0.024	0.088
Research Fellow, Research Associate	−1.838***	0.104	−1.743***	0.089	−1.799***	0.103	−1.750***	0.089
Lecturer	−1.298***	0.089	−1.883***	0.080	−1.295***	0.089	−1.883***	0.080
Reader, Associate Professor, Senior Lecturer	−0.718***	0.071	−1.204***	0.063	−0.717***	0.071	−1.202***	0.063
User-inspired basic research	1.669***	0.083	1.031***	0.066	1.670***	0.082	1.038***	0.066
Applied research	2.335***	0.076	1.341***	0.064	2.318***	0.076	1.360***	0.064
None of the above apply to my research	0.558***	0.165	−0.179	0.176	0.537***	0.164	−0.161	0.176
Career Motivation: Intrinsic	1.053***	0.062	0.890***	0.054	1.058***	0.062	0.878***	0.054
Research Council Funding	0.705***	0.079	0.277***	0.063	0.670***	0.078	0.290***	0.063
Life Science & Health	0.278***	0.068	0.117*	0.062	0.279***	0.067	0.109*	0.062
Arts and Humanities	0.058	0.087	−0.229***	0.084	0.022	0.087	−0.222***	0.084
Engineering, Maths, Physics	0.296***	0.080	0.384***	0.066	0.382***	0.080	0.339***	0.066
Post 1992 University	0.142	0.097	−0.115	0.096	0.122	0.097	−0.103	0.096
Russell Group University	−0.136*	0.079	−0.327***	0.064	−0.148*	0.079	−0.322***	0.064
University REF Research Output Score	−0.299***	0.084	−0.034	0.098	−0.281***	0.082	−0.044	0.098
University research contract income	0.048	0.047	0.339***	0.044	0.042	0.047	0.341***	0.044
NUTS2_R&D expenditure (2014)	0.000	0.003	0.012***	0.003	−0.001	0.003	0.012***	0.003
NUTS3_Population Density (2015)	−0.054***	0.020	0.026	0.017	−0.047**	0.020	0.023	0.017
region NORTHERN IRELAND	0.449**	0.175	0.351**	0.150	0.349**	0.173	0.393***	0.150
region SCOTLAND	0.178**	0.088	0.013	0.074	0.177**	0.088	0.010	0.074
region WALES	0.109	0.115	−0.355***	0.118	0.112	0.115	−0.354***	0.118
Observations	14574		14574		14574		14574	
Pseudo R-square	0.0868		0.1237		0.0890		0.1244	

Marginal effects and robust standard errors are reported; Reference categories: UK born, Age 50∼, Professor, Social Sciences, England University (other)); * p < 0.10, ** p < 0.05, *** p < 0.01.

As expected, foreign-born academics have a smaller intranational engagement breadth, but a larger international breadth compared to UK-born academics thus confirming Hypotheses 1a and 1b. In Model 1, we find that they engage through 0.6 fewer intranational activities, which is 12 per cent less than their UK-born colleagues. Instead, they engage through one additional international activity, which is a 40 per cent increase compared to their UK-born average peers. Model 2 shows that this negative correlation on intranational activity diminishes the longer foreign-born staff have worked in the UK in line with Hypothesis 2a. Thus, while *recent arrivals* still engage through 18 per cent fewer activities intranationally compared to UK-born, this difference is diminished to less than one per cent and becomes insignificant for *settled* foreign-born academics when we control for language and ethnicity. Returnees, i.e. those who were born in the UK but spent a period abroad, show a lower intranational engagement breadth of 11 per cent compared to those who have never been abroad, but have a higher level of international engagement of about 0.7 activities or 28 per cent. While the difference between returnees and foreign-born does not vanish completely (i.e. foreign-born still engage through more activities internationally), it is significantly reduced, providing support to Hypothesis 2b. The results also show that the international advantage of the foreign-born persists, even after having remained in the host country for an extended period of time.

We further find that a non-white ethnicity of the foreign-born academic further increases the differences to UK-born in terms of intranational engagement in line with Hypothesis 3a. UK-born academics of a non-white ethnic background show a higher breadth of international engagement compared to the white UK population but not to the extent of foreign-born, thus providing only limited evidence for Hypothesis 3b. Finally, coming from a non-English speaking country has a strong negative correlation with intranational engagement and a positive correlation with international engagement activity. This heightened difference between native and foreign-born provides support for Hypotheses 4a and 4b.

To summarise, the results show that those that are new to the UK and those that have English as their second or third language show lower levels of intranational engagement. New arrivals or those from non-English speaking countries engage on average one activity less than their UK-born colleagues. This means that while the UK-born engage on average through five activities, these groups engage through four and thus 20 per cent less. Instead, new arrivals engage through 1.3 (or 50 per cent) more international activities and settled academics still through 0.9 (or 35 per cent) more compared to their non-mobile UK-born peers.

In general, the controls are consistent across the two models, except for PhD and employment years that are also used to build the experience variables. We find that women engage through fewer activities in both intra- and international contexts, but the effect is larger in the international context. Engagement increases with seniority in both contexts with the effects being greater for international engagement. Engagement activity is lowest for those younger than 40, and intranational activities are highest for those aged 40–49. A PhD in the UK is positively associated with intranational engagement and negatively with international engagement but becomes weaker once experience measures are included. We also find a positive sign for more applied types of research, research council funding and for higher intrinsic career motivations and find that all of these effects are stronger in an intranational context. A wider engagement breadth is found for engineering and the life sciences, both within the intra- and international context. Higher quality institutions generally show lower engagement breadth. We also find more engagement in the devolved regions of Northern Ireland and Scotland. In general, these results are consistent with the prior literature on academic engagement (Perkmann et al., 2013).

### 7.3 Average treatment effect results

To further explore these results, we turn to the matching approach, as there may be underlying differences between foreign and UK-born academics that bias our results. Table 5 reports the engagement breadth of foreign-born and their matched UK-born counterparts as well as the average treatment effect of the treated for all academics and for each sub-group of experience, ethnicity and native language. The average treatment effect of the treated (ATT) column reports the differences in mean breadth of interactions between measures of academic foreignness and the UK-born. Differences in each of the subsets of the foreign-born are based on comparisons with their matched UK-born pair and not all UK-born academics. Equally, average differences in each subset of UK-born are based on comparisons with their next foreign-born neighbour.

**Table 5:** Difference in engagement between foreign and UK born academics after matching (max 15).

		intranational				international			
	Obs	UK-born	Foreign			UK-born	Foreign		
	(per group)	mean	mean	ATT	SE	mean	mean	ATT	SE
Foreign-born‡	1451	4.921	4.477	−0.445^***^	(0.145)	2.393	3.343	0.950^***^	(0.125)

Characteristics of UK born†									
Returnee	194	3.593	3.366	−0.227	(0.478)	2.423	3.314	0.892^**^	(0.390)
Non-white UK born	56	4.714	2.964	−1.750^**^	(0.822)	3.071	2.696	−0.375	(0.661)
White stayers	1219	5.119	4.684	−0.435^***^	(0.151)	2.357	3.355	0.998^***^	(0.132)

Characteristics of foreign born‡									
Time in UK = recent	89	3.27	2.427	−0.843	(0.756)	2.022	2.764	0.742	(0.522)
Time in UK = settled	599	4.384	3.886	−0.497^**^	(0.206)	2.040	2.688	0.648^***^	(0.161)
Time in UK = long-term settled	763	5.536	5.180	−0.356*	(0.189)	2.713	3.924	1.211^***^	(0.177)
Non-white foreign born	416	4.887	4.173	−0.714^***^	(0.245)	2.361	3.435	1.075^***^	(0.214)
Non English native	902	4.885	4.013	−0.871^***^	(0.181)	2.313	3.365	1.052^***^	(0.153)

* p < 0.10, ** p < 0.05, *** p < 0.01. Lechner-adjusted standard errors in parentheses (see Lechner, 2001).

‡ Difference compared to matched UK born academic.

† Difference of matched foreign born academic.

The ATT results confirm Hypotheses 1a and 1b as well as the results from the regression showing that the foreign-born engage through significantly fewer activities with intranational actors but more with international actors compared with their native-born match. To illustrate, the observed difference is 0.45 (10 per cent) in terms of intranational engagement breadth and 0.95 (40 per cent) in terms of international breadth and thus similar to the Poisson estimates. We also confirm that differences in intranational engagement diminish with time spent in the UK. This result is consistent with our Hypothesis 2a. We find that returnees engage less internationally than their matched foreign-born pair thus rejecting Hypothesis 2b. We find stronger support for Hypotheses 3a and 3b than we found in the first part of our analysis. Specifically, the difference in intranational engagement between native and foreign-born academics is larger for a foreigner of a non-white ethnic background. Also, the difference in international engagement between native and foreign-born is diminished for native academics of non-white ethnicity. The matching again confirms the lower intranational engagement for those with English as a second or third language, which is consistent with Hypotheses 4a. They engage through 0.9 (or 17 per cent) fewer activities. International engagement is also slightly heightened, but the difference to the baseline (ATT for all native and foreign-born) is very small, at just three per cent.

## 8 Supplementary analysis

In this section we check in several robustness regressions the sensitivity of our results to constructions of our key variables. First, recall that although the survey enquired about 27 types of engagement, we reduced this to fifteen for our analysis. Here we test if results hold for all 27 types as well as for a more limited group of just five selected activities which offer engagement that is project and/or research based: a) joint research with external organisations; b) participating in research consortia; c) contract research, d) consultancy services; and e) providing informal advice. In the case of 27 channels the mean number of activities undertaken is 6.4 intranationally (7.0 for UK-born and 5.2 for foreign-born) and 3.2 internationally (2.8 for UK-born and 3.9 for foreign-born). Also, in the case of a reduced set of five channels, we continue to observe differences between UK and foreign-born: 1.6 versus 1.2 in the case of intranational engagement activities and 0.7 versus 1.0 in the case of international activities. Using this descriptive information, we repeat our estimations from above. Results of the treatment effect estimation are reported in Table 6 and confirm previous results with the strongest home effect found for new arrivals and those from non-English speaking countries.^3^

**Table 6:** Difference in engagement between foreign and UK born academics after matching (for broader and narrower selection of engagement activities).

		27 activities		5 activities	
	Obs	intranational	international	intranational	international
	(per group)	ATT	ATT	ATT	ATT
Foreign-born‡	1451	−0.513^***^	1.160^***^	−0.180^***^	0.276^***^
		(0.186)	(0.147)	(0.060)	(0.051)

Characteristics of UK born†					
Returnee	194	−0.469	1.010^**^	−0.062	0.294^*^
		(0.621)	(0.456)	(0.196)	(0.166)
Non-white UK born	56	−2.250^**^	−0.411	−0.786^**^	−0.232
		(1.095)	(0.854)	(0.371)	(0.354)
White stayers	1219	−0.461^**^	1.231^***^	−0.175^***^	0.290^***^
		(0.194)	(0.156)	(0.063)	(0.054)

Characteristics of foreign born‡					
Time in UK = recent	89	−1.045	0.876	−0.236	0.213
		(0.972)	(0.615)	(0.313)	(0.212)
Time in UK = settled	599	−0.566^**^	0.758^***^	−0.160^*^	0.175^**^
		(0.255)	(0.186)	(0.088)	(0.070)
Time in UK = long-term settled	763	−0.409^*^	1.509^***^	−0.189^**^	0.363^***^
		(0.248)	(0.210)	(0.078)	(0.071)
Non-white foreign born	416	−0.793^**^	1.370^***^	−0.344^***^	0.243^***^
		(0.314)	(0.256)	(0.102)	(0.087)
Non English native	902	−1.014^***^	1.283^***^	−0.309^***^	0.349^***^
		(0.231)	(0.182)	(0.076)	(0.063)

* p < 0.10, ** p < 0.05, *** p < 0.01. Lechner-adjusted standard errors in parentheses (see Lechner, 2001). Set of five activities include: a) joint research with external organisations; b) participating in research consortia; c) contract research, d) consultancy services; and e) providing informal advice.

‡ Difference compared to matched UK born academic.

† Difference of matched foreign born academic.

Second, to understand whether these differences in the geography of academic engagement were related to local and regional engagement, we conducted an additional analysis. Table 7 shows differences for local (within 10 miles) and regional (within NUTS1) activities. The mean number of local activities undertaken is 1.9 (2.0 for UK-born and 1.6 for foreign-born) and the mean number of regional activities (including local) is 3.2 (3.4 for UK-born and 2.7 for foreign-born). The treatment effect models confirm the negative foreign-born effect, but the marginal effect is weaker compared to intranational activities due to the lower number of overall activities undertaken. The differences between regional (NUTS1) and intranational engagement are small and results consistent across the two levels of analysis. Locally, the difference between foreign and native born disappear with the one exception being the negative sign for those from a non-native English language background. One potential reason for observing fewer differences at the local level may be that the differences between the foreign- and native-born manifest themselves more strongly at the national level, as this is where the cultural and institutional differences are rooted. Foreign-born academics may also, through living and working in their local environments, build up social capital at a local level more easily than at a regional or national level. However, further work is required to better understand the salience of different geographic proximity variables for academic engagement.

**Table 7:** Difference in engagement between foreign and UK born academics after matching (for engagement activities at the Local and Regional level).

			15 activities			
	Obs	Local (<10 miles)			Region (NUTS1)	
	(per group)	ATT		SE	ATT	SE
Foreign-born‡	1451	0.014		(0.098)	−0.236*	(0.122)

Characteristics of UK born†						
Returnee	194	−0.010		(0.250)	−0.16	(0.326)
Non-white UK born	56	−0.143		(0.486)	−1.018	(0.701)
White Stayers	1219	0.024		(0.107)	−0.212	(0.130)

Characteristics of foreign born‡						
Time in UK = recent	89	0.056		(0.347)	−0.056	(0.454)
Time in UK = settled	599	−0.145		(0.142)	−0.381^**^	(0.180)
Time in UK = long-term settled	763	0.134		(0.134)	−0.143	(0.161)
Non-white foreign born	416	−0.118		(0.154)	−0.483^**^	(0.198)
Non English native	902	−0.233*		(0.114)	−0.629^***^	(0.147)

* p < 0.10, ** p < 0.05, *** p < 0.01. Lechner-adjusted standard errors in parentheses (see Lechner, 2001).

‡ Difference compared to matched UK born academic.

† Difference of matched foreign born academic.

## 9 Conclusions

This study demonstrates that foreign and native-born academics differ in their geography of academic engagement, with foreign-born academics looking relatively more outwards toward international actors, and native-born looking relatively more inwards towards national actors. Foreign-born academics appear to demonstrate a ‘liability of foreignness’ when it comes to intranational engagement, and native-born academics have a ‘liability of domesticity’ when it comes to international engagement. These differences are robust to comparisons between individuals working at the same university, rank and discipline. It should be noted, however, that these differences are modest, and many foreign-born academics do engage with national actors and native-born academic do engage with international actors. In particular, foreign-born academics often exhibit *both* high levels of intranational and international engagement, contrary to any suggestion that they are ‘citizens of nowhere’ in their professional roles. Moreover, we find some evidence that native-born academics benefit from migration experience in terms of encouraging international engagement.

These results suggest that by engaging with international actors, foreign-born academics are relatively more likely to act as a conduit to international contacts. This idea is consistent with Edler et al. (2011), who show that international scientific mobility spurs engagement with both national and foreign firms. In addition, by helping to ensure that the national science system is focused on global challenges rather than simply local needs, their presence may help to increase the absorptive capacity of the science system. Through their external engagements, foreign-born academics may also help to amplify the global influence of the national institutions of which they are members. It could be argued that one of the reasons UK universities consistently score highly on international rankings, which are based on surveys of influential international actors, is that they able to draw on the goodwill generated by the large, engaged cohort of foreign-born staff (Lepori et al., 2015). Moreover, it is likely that the international engagement efforts of foreign-born academics generate spillovers for native-born academics, helping them to align their research efforts to more international challenges and opportunities. The same effect may also be true for the native-born, who act as ‘anchors’ to facilitate engagement with intranational actors. In doing so, native-born academics may provide a channel to bring in international knowledge and experience to tackle national challenges, providing a bridge between needs and problems arising within the national context and with ideas and solutions found elsewhere.

Our study also suggests that personal experience and factors matter when looking at the geography of academic engagement. Critically, we demonstrate that the differences between foreign and native-born academics in intranational engagement tend to fade out as the foreign-born academics spend greater time in the UK. This suggests with sufficient experience in the domestic context, foreign-born academics will demonstrate the same degree of ‘citizenship’ in terms of local engagement as their native-born colleagues. Moreover, we found that native-born academics with foreign experience were more likely than their native-born colleagues to be active in international engagement. This indicates that native-born academics may benefit from international mobility, gaining new experiences and relationships that can be useful in amplifying their efforts when they return to their home country (Edler et al., 2011; Gibson and McKenzie, 2014).

The research also demonstrates that ethnic background and language skills might play a role in shaping the geography of academic engagement. In particular, we showed that non-majority foreign-born academics had lower levels of intranational engagement than majority foreign-born academics. Moreover, native-born academics with non-majority ethnic profiles were more likely to engage internationally than their majority native-born colleagues. This suggests that ethnic diversity within the university may help to increase international engagement, as individuals are able to draw upon cultural and institutional knowledge to help foster relationships with international actors. Accordingly, the rich diversity among foreign staff at UK universities might provide a strong resource from which to build up the UK’s reputation as a ‘beacon of openness’. An additional implication of these findings is that non-majority academics – foreign-born and native-born - may find it more challenging to form intranational relationships, especially in contexts where there is limited diversity. We also show that language skills matter, as foreign-born academics from non-English speaking countries are less widely engaged intranationally.

Our results are, however, of interest beyond the UK since the internationalisation of academic staff is a pervasive feature of universities in other major economies (Lepori et al., 2015). Thus, in 2011, it has been estimated in a sample of Natural Science and Engineering disciplines that foreign-born researchers accounted for over 50 per cent of publishing researchers in Switzerland, 38 per cent in USA and Sweden, 28 per cent in Netherlands and 23 per cent in Germany (Franzoni et al., 2012). The UK is a good context for this study as we can largely ignore border effects in international engagement, something that may be very different for the aforementioned national contexts.

There are several policy implications that emerge from this work. First, although there is an expectation in much of the literature that engagement is a positive activity for both academics and external actors, it is not clear that all members of the academic community are equally placed to be effective in this role. In particular, foreign-born academics, often operating in their second language and often part of a non-majority ethnic group, may face greater barriers to intranational engagement than native-born academics, perhaps more especially at regional and national levels as opposed to the local level. At the same time, they may be more effective at international engagement. Currently, the literature on academic engagement has given modest attention to the geography of these engagements, and many of the policy initiatives encouraging academics to engage have been ‘place-free’. For instance, in the UK’s recent REF assessment, the required case studies of impact had no geographical restriction, and the ‘reach and significance’ of impact could have been achieved within or outside the UK (HEFCE, 2015). The issue for consideration for universities and for national assessments is to what extent there should be equal rewards and appreciation for intranational or international engagement by academic faculty. One could make a ‘nativist’ argument that these systems should favour intranational engagement, even at the cost of international engagement, to ensure that the benefits associated with academic research are more likely to spillover in the country where these academic efforts take place. Indeed, it could be suggested that such an approach would be an antidote to the ‘extra-territorial’ nature of science. However, such an approach would assume that local engagement efforts are themselves immune from international ones, which is unlikely to be the case. Moreover, international engagement is liable to help to increase the ‘reach’ and ‘significance’ of national research by connecting to global pipelines of knowledge and resources, as well as enhancing its potential to act as a ‘beacon of openness’.

Second, given that there are now increasing incentives and rewards for academics to engage, especially with national actors, foreign-born academics operating in an environment distant from their own might find it hard to achieve these objectives. Even in the case of equal treatment for intra- and international engagement, proactive measures and training may be necessary for foreign-born academics to establish and build these contacts with national actors. This suggests that care must be given to ensure that rewards systems at universities, especially for junior staff, do not expose foreign-born academics to systemic disadvantages.

Third, our study shows that native-born academics that have worked or have education experience abroad are more effective at international engagement. This suggests that encouraging international mobility of native-born academic staff may help to build their capacity to find partners from outside their home context, and therefore increase the international reach and significance of their work. Greater efforts to spur native-born academics to work abroad might help to influence the nature of their external engagement. This suggests that ensuring high international mobility by academics, often financed through international research collaboration programmes, can be an important spur to future international engagement with non-academic actors (Edler et al., 2011).

### 9.1 Limitations and future research

There are several significant limitations to our study, which in turn open a range of questions for future research. First, although we have rich information about individuals’ engagement efforts across different channels, we have given little attention to the frequency of these engagements in each channel. It may be that some individuals engage with multiple actors in each channel, such as having multiple industry partners for collaborative research. As such, our measure reflects the geography of engagement breadth rather than geography of engagement depth. The measure also does not take into account the relevance or effectiveness of each engagement channel in the intra- and international context, but assumes that there is no difference. Future research should investigate the importance of each channel for different places. Our study also has no information on the country of international engagement, thus throwing up the question as to the place-based nature of these activities. For instance, while we may expect a foreign Mexican academic to be more likely to collaborate with firms in Mexico than a native UK academic, it is not clear whether we would expect them to differ in their engagement with (for example) partners in Korea. Lacking this information, we are not able to answer the question whether foreign-born possess a different ‘mindset’ or different social capital compared to native-born. In the case of scientific collaborations, Scellato et al. (2015) show that links to country of origin and to a diaspora correlate strongly with network size, and conclude that networks are portable. Based on their findings, we cannot rule out that the origin effect is a likely explanation for the higher international engagement, which requires a more detailed study of the context of external engagement.

Second, as it stands, we lack complete information on the career pathways of academics in our sample. It may be that some UK-born academics have greater international exposure than we have accounted for in our measures. Moreover, it may be that foreign-born academics are themselves now UK citizens and/or have a UK partner, and therefore have a greater degree of attachment to the national context. At this stage, we are unable to say whether our results are partly driven by unobserved mobility decisions or by the degree of attachment that individuals feel to their national environment. Further research should develop rich career histories of academics to help better understand how career experiences within and outside their home country shape the nature of their engagement efforts. This research could also explore the degree of attachment of individuals to their national context or, even more narrowly, their local ‘place’ and how that shapes their attitudes and behaviours with regards to engagement. At present, we are unable to explore the possibility that more locally oriented foreign-born academics decide to remain in the UK, while those with a non-host country orientation may decide to move on or return to their home country.

Third, since our research focuses on UK-based academics, there is a danger that our research results cannot be generalized to national settings, where presence of foreign-born nationals within the academic sector is unusual. It may be that in academic systems with lower levels of internationalization, such as Portugal or Japan, the patterns observed would be very different. For example, in less international systems, the differences between foreign and native-born in terms of engagement with non-academic actors may be heightened. Future research should examine how national context influences behaviour of foreign and native-born academics in their engagement with non-academic actors.

Fourth, although we have attempted to take account of university and regional context and we also attempt to match foreign- and native-born academics at the same university, age and department, our focus has been primarily at the individual level. It may be that it is the features of the university and/or regional context that shape the engagement behaviours of foreign-born or native-born academics. For example, a diverse, tolerant local environment might mitigate differences between foreign and native-born academics. Alternatively, a regional context with many international firms or organizations might facilitate international engagement for native-born academics. It would be useful to explore the sub-national ‘place-based’ factors that give rise to these differences with greater detail and attention.

Fifth, we have speculated that connections between foreign and native-born academics might be a mechanism to enable productive combinations of national and international problems. However, we have not investigated this issue in any detail and future research could explore the effect of employment of foreign-born staff on the engagement efforts of native-born (and vice-versa). It could also explore the potential of international scientific collaborations by native-born academics as means to enable or enrich their non-local engagement efforts. We can also only speculate about the spatial research orientation of the foreign and native-born, and future research should investigate whether native-born engage in more place-based research themes compared to foreign-born, which may explain some of the differences observed here.

Despite these limitations, this study has helped to bring attention to the geography of academic engagement, exposing how where people were born and where they have worked shape whom they engage with. In doing so, we hope to have enriched our understanding of the ‘citizenship’ of academics with national and international external actors.

## Funding

The authors acknowledge support from the Arts and Humanities Research Council, the Department for Business, Innovation & Skills, the Economic and Social Research Council, the Engineering and Physical Sciences Research Council, the Higher Education Funding Council for England, the Medical Research Council, and the Natural Environment Research Council and the National Centre for Universities and Business (NCUB). Cornelia Lawson received support from the University of Bath through a Prize fellowship.

## Figures and Tables

**Table A1:** Prediction of treatment (being foreign) before and after matching.

	BEFORE		AFTER	
	FOREIGN		FOREIGN	
Basic research	0.309^***^	(0.065)	0.072	(0.064)
User-inspired basic research	0.426^***^	(0.065)	−0.052	(0.059)
Applied research	0.220^***^	(0.063)	−0.200	(0.158)
Research Council Funding	−0.173^***^	(0.039)	−0.109	(0.076)
PHD in UK	−1.877^***^	(0.035)	0.008	(0.086)
AGE <40	0.259^***^	(0.030)	−0.007	(0.057)
AGE >50	−0.378^***^	(0.03)	0.060	(0.064)
_cons	0.936^***^	(0.070)	−0.001	(0.097)
Observations	14811		2902	
Log-Likelihood	−7195.183		−2006.976	
Pseudo R-square	0.253		0.002	

Standard errors in parentheses; * p < 0.10, ** p < 0.05, *** p < 0.01. Propensity score matching is combined with elements of an exact matching (EM) procedure on academic rank, disciplinary field, university and gender.

## Footnotes

1 There are a handful of typically small “private” universities outside this system with a primary focus on teaching.

2 We test the representativeness of our sample in terms of external activities and research direction by comparing those academics who replied without a reminder with those academics who required prompting to respond, and those that completed the survey with those that left incomplete responses. The findings suggest that differences are small and insignificant when we control for demographic characteristics. In terms of response distribution, we find a slight underrepresentation in the arts and humanities, possibly due to a difficulty in reaching out to those lecturing in the performing and practicing arts primarily at art or music schools. Overall our response bias tests give us confidence that our results are representative.

3 Results of the Poisson again confirm the main findings and are available from the authors upon request.
